# The genome sequence of the bronze furrow bee, *Seladonia tumulorum *(Linnaeus, 1758)

**DOI:** 10.12688/wellcomeopenres.17784.1

**Published:** 2022-03-21

**Authors:** Steven Falk, University of Oxford and Wytham Woods Genome Acquisition Lab, Darwin Tree of Life Barcoding collective, Wellcome Sanger Institute Tree of Life programme, Wellcome Sanger Institute Scientific Operations: DNA Pipelines collective, Tree of Life Core Informatics collective, Joseph Monks, Darwin Tree of Life Consortium

**Affiliations:** 1 Independent Researcher, Kenilworth, Warwickshire, UK; 2 Department of Life Sciences, Natural History Museum, London, UK

**Keywords:** Seladonia tumulorum, bronze furrow bee, genome sequence, chromosomal, Hymenoptera

## Abstract

We present a genome assembly from an individual male *Seladonia tumulorum* (the bronze furrow bee; Arthropoda; Insecta; Hymenoptera; Halictidae). The genome sequence is 479 megabases in span. The majority of the assembly (84.28%) is scaffolded into 17 chromosomal pseudomolecules. The mitochondrial genome was also assembled and is 17.3 kilobases in length.

## Species taxonomy

Eukaryota; Metazoa; Ecdysozoa; Arthropoda; Hexapoda; Insecta; Pterygota; Neoptera; Endopterygota; Hymenoptera; Apocrita; Aculeata; Apoidea; Anthophila; Halictidae; Halictinae; Halictini; *Halictus (Seladonia) tumulorum* (Linnaeus, 1758) (NCBI:txid2795680).

## Background

*Halictus (Seladonia) tumulorum* (bronze furrow bee) is a small (forewing length 4–5.5 mm in length) metallic species common throughout the UK although becoming scarcer in Scotland ( Benton, 2017). The species is present across the Palearctic from the UK to Japan. Females utilise sallows in the early spring. The species is very close in appearance to *H. (Seladonia) confusus* Smith and can only be confidently separated by examining the male genitalia. The species is primitively eusocial and has been recorded as exhibiting weak polyphenism: the foundress and workers appear similar and overlap in size (Plateaux-Quénu and Plateaux 1994). Females are active from March, while males emerge later in June.

## Genome sequence report

**Figure 1.**
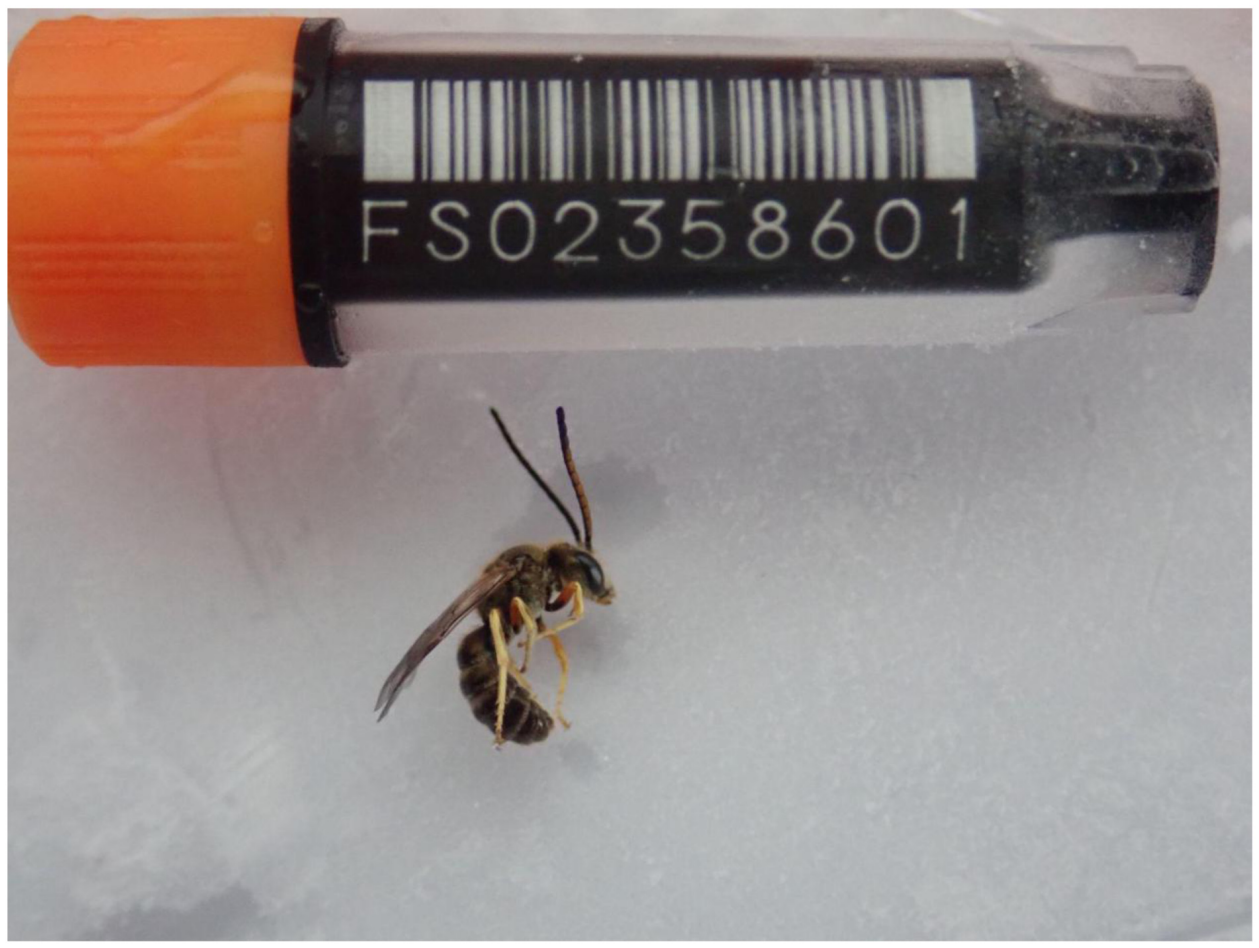
Image of the iySelTumu1 specimen taken during preservation and processing.

The genome was sequenced from a single male *S. tumulorum* ( Figure 1) collected from Wytham Woods, Oxfordshire (biological vice-county: Berkshire), UK (latitude 51.767, longitude -1.311). A total of 58-fold coverage in Pacific Biosciences single-molecule long reads and 93-fold coverage in 10X Genomics read clouds were generated. Primary assembly contigs were scaffolded with chromosome conformation Hi-C data. Manual assembly curation corrected 19 missing/misjoins, increasing the assembly size by 7.59%, the scaffold number by 196.08% and the scaffold N50 by 21.46%.

**Figure 2.**
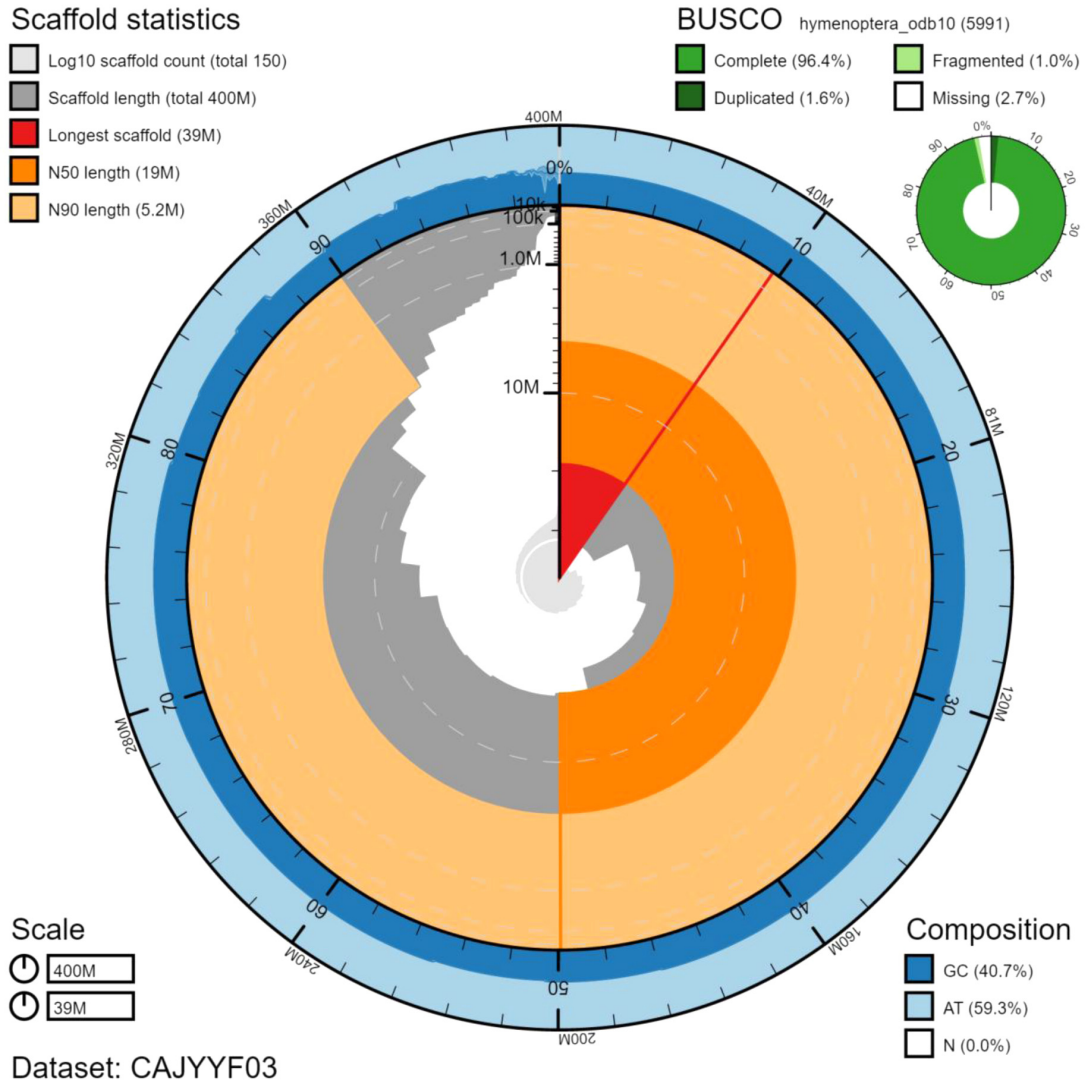
Genome assembly of *Seladonia tumulorum*, iySelTumu1.3: metrics. The BlobToolKit Snailplot shows N50 metrics and BUSCO gene completeness. The main plot is divided into 1,000 size-ordered bins around the circumference with each bin representing 0.1% of the 404,441,066 bp assembly. The distribution of scaffold lengths is shown in dark grey with the plot radius scaled to the longest scaffold present in the assembly (39,298,666 bp, shown in red). Orange and pale-orange arcs show the N50 and N90 scaffold lengths (18,583,095 and 5,248,087 bp), respectively. The pale grey spiral shows the cumulative scaffold count on a log scale with white scale lines showing successive orders of magnitude. The blue and pale-blue area around the outside of the plot shows the distribution of GC, AT and N percentages in the same bins as the inner plot. A summary of complete, fragmented, duplicated and missing BUSCO genes in the hymenoptera_odb10 set is shown in the top right. An interactive version of this figure is available at https://blobtoolkit.genomehubs.org/view/iySelTumu1.3/dataset/CAJYYF03/snail.

The final assembly has a total length of 404 Mb in 151 sequence scaffolds with a scaffold N50 of 18.6 Mb ( Table 1). Of the assembly sequence, 84.28% was assigned to 17 chromosomal-level scaffolds (numbered by sequence length) ( Figure 2– Figure 5; Table 2). Hi-C associations between chromosome arms were very faint, leaving some room for potentially missing joins. No existing karyotype information was found, leaving a small amount of uncertainty over the final number of chromosomes that were identified. The assembly has a BUSCO v5.2.2 ( Manni *et al*., 2021) completeness of 96.4% (single 94.8%, duplicated 1.6%) using the hymenoptera_odb10 reference set (n=5991).

**Table 1.** Genome data for *Seladonia tumulorum*, iySelTumu1.3.

*Project accession data*	
Assembly identifier	iySelTumu1.3
Species	*Seladonia tumulorum*
Specimen	iySelTumu1 (male, genome assembly); iySelTumu2 (male, Hi-C)
NCBI taxonomy ID	NCBI:txid115100
BioProject	PRJEB45675
BioSample ID	SAMEA7746445
Isolate information	Whole organisms
*Raw data accessions*	
PacificBiosciences SEQUEL II	ERR6807989
10X Genomics Illumina	ERR6363323-ERR6363326
Hi-C Illumina	ERR6363322
*Genome assembly*	
Assembly accession	GCA_913789895.3
Span (Mb)	404
Number of contigs	163
Contig N50 length (Mb)	15.0
Number of scaffolds	151
Scaffold N50 length (Mb)	18.6
Longest scaffold (Mb)	39.3
BUSCO * genome score	C:96.4%[S:94.8%,D:1.6%], F:1.0%,M:2.7%,n:5991

*BUSCO scores based on the hymenoptera_odb10 BUSCO set using v5.2.2. C= complete [S= single copy, D=duplicated], F=fragmented, M=missing, n=number of orthologues in comparison. A full set of BUSCO scores is available at https://blobtoolkit.genomehubs.org/view/iySelTumu1.3/dataset/CAJYYF03/busco.

**Figure 3.**
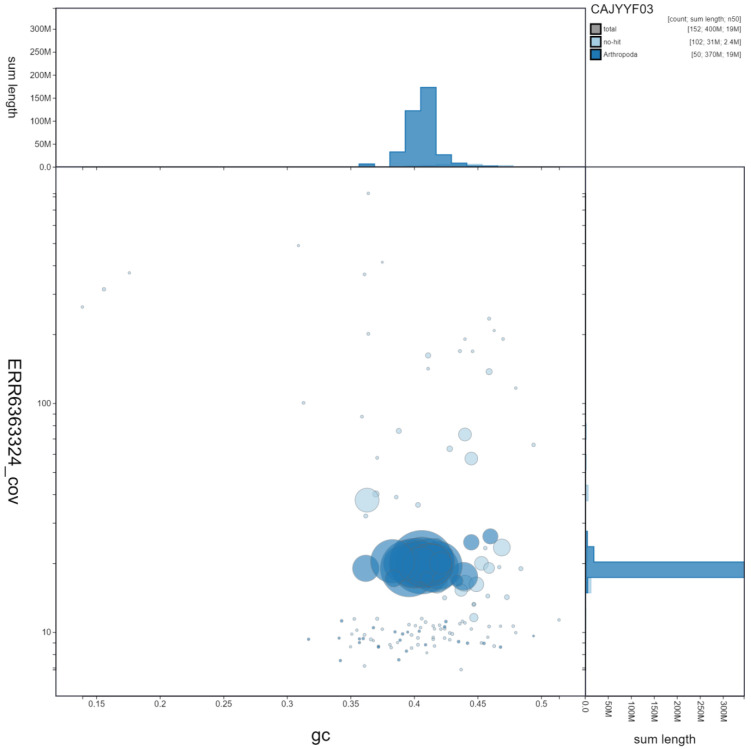
Genome assembly of *Seladonia tumulorum*, iySelTumu1.3. GC coverage. BlobToolKit GC-coverage plot. Scaffolds are coloured by phylum. Circles are sized in proportion to scaffold length. Histograms show the distribution of scaffold length sum along each axis. An interactive version of this figure is available at https://blobtoolkit.genomehubs.org/view/iySelTumu1.3/dataset/CAJYYF03/blob.

**Figure 4.**
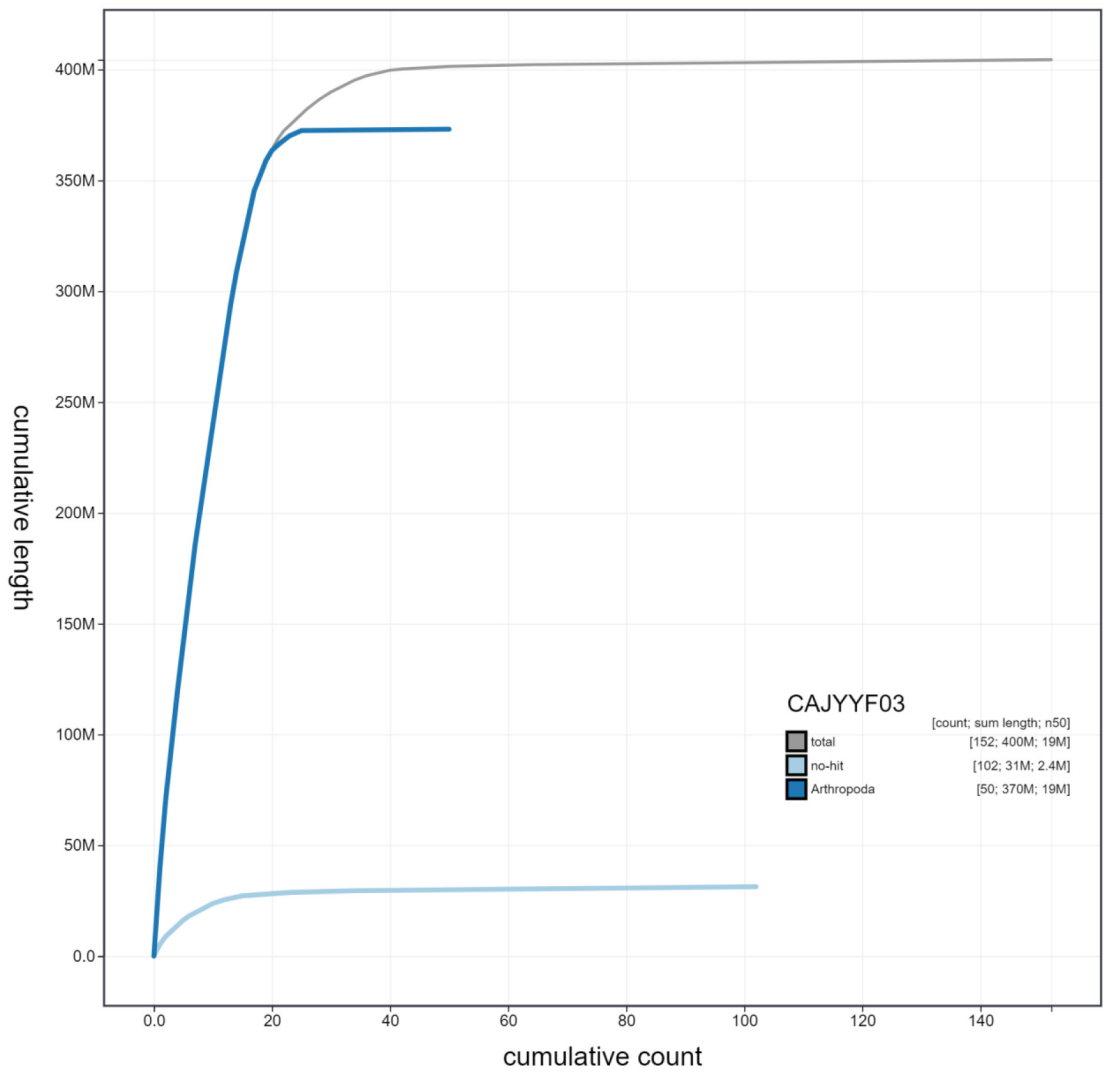
Genome assembly of *Seladonia tumulorum*, iySelTumu1.3: cumulative sequence. BlobToolKit cumulative sequence plot. The grey line shows cumulative length for all scaffolds. Coloured lines show cumulative lengths of scaffolds assigned to each phylum using the buscogenes taxrule. An interactive version of this figure is available at https://blobtoolkit.genomehubs.org/view/iySelTumu1.3/dataset/CAJYYF03/cumulative.

**Figure 5.**
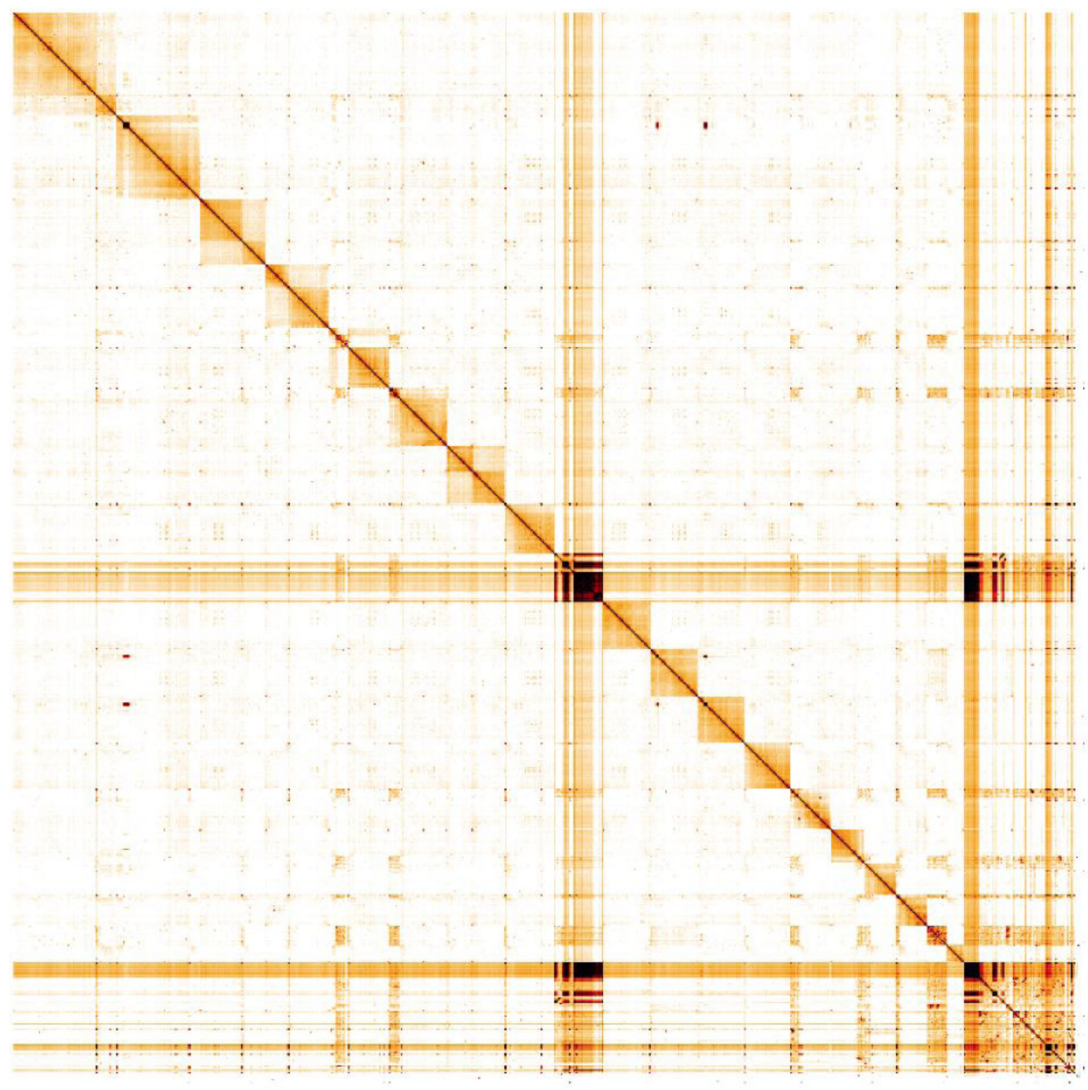
Genome assembly of *Seladonia tumulorum*, iySelTumu1.3: Hi-C contact map. Hi-C contact map of the iySelTumu1.3 assembly, visualised in HiGlass. Chromosomes are shown in size order from left to right and top to bottom. An interactive version of this map is available here.

**Table 2.** Chromosomal pseudomolecules in the genome assembly of *Seladonia tumulorum*, iySelTumu1.3.

INSDC accession	Chromosome	Size (Mb)	GC%
OU565266.1	1	39.30	40.6
OU565267.1	2	31.56	39.6
OU565268.1	3	17.84	40.3
OU565269.1	4	24.79	40.6
OU565270.1	5	24.02	40.0
OU565271.1	6	22.42	41.6
OU565272.1	7	21.96	41.9
OU565273.1	8	21.92	41.3
OU565274.1	9	18.58	39.9
OU565275.1	10	18.16	40.4
OU565276.1	11	17.58	40.8
OU565277.1	12	17.46	41.3
OU565278.1	13	15.18	41.2
OU565279.1	14	12.77	41.5
OU565280.1	15	11.87	39.0
OU565281.1	16	11.77	40.5
OU565282.1	17	6.44	36.2
OU565283.1	MT	0.02	17.9
-	Unplaced	70.82	41.3

## Methods

### Sample acquisition and DNA extraction

Two male *S. tumulorum* specimens (iySelTumu1 and iySelTumu2) were collected from Wytham Woods, Oxfordshire (biological vice-county: Berkshire), UK (latitude 51.767, longitude -1.311) by Steven Falk, Independent Researcher, using a net. The samples were identified by the same individual and snap-frozen on dry ice.

DNA was extracted at the Tree of Life laboratory, Wellcome Sanger Institute. The iySelTumu1 sample was weighed and dissected on dry ice. Whole organism tissue was disrupted using a Nippi Powermasher fitted with a BioMasher pestle. Fragment size analysis of 0.01–0.5 ng of DNA was then performed using an Agilent FemtoPulse. High molecular weight (HMW) DNA was extracted using the Qiagen MagAttract HMW DNA extraction kit. Low molecular weight DNA was removed from a 200-ng aliquot of extracted DNA using 0.8X AMpure XP purification kit prior to 10X Chromium sequencing; a minimum of 50 ng DNA was submitted for 10X sequencing. HMW DNA was sheared into an average fragment size between 12–20 kb in a Megaruptor 3 system with speed setting 30. Sheared DNA was purified by solid-phase reversible immobilisation using AMPure PB beads with a 1.8X ratio of beads to sample to remove the shorter fragments and concentrate the DNA sample. The concentration of the sheared and purified DNA was assessed using a Nanodrop spectrophotometer and Qubit Fluorometer and Qubit dsDNA High Sensitivity Assay kit. Fragment size distribution was evaluated by running the sample on the FemtoPulse system.

### Sequencing

Pacific Biosciences HiFi circular consensus and 10X Genomics read cloud sequencing libraries were constructed according to the manufacturers’ instructions. Sequencing was performed by the Scientific Operations core at the Wellcome Sanger Institute on Pacific Biosciences SEQUEL II and Illumina NovaSeq 6000 instruments. Hi-C data were generated from whole organism tissue of iySelTumu2 using the Arima v2.0 kit and sequenced on an Illumina NovaSeq 6000 instrument.

### Genome assembly

Assembly was carried out with Hifiasm ( Cheng *et al*., 2021). Haplotypic duplication was identified and removed with purge_dups ( Guan *et al*., 2020). Scaffolding with Hi-C data ( Rao *et al*., 2014) was carried out with SALSA2 ( Ghurye *et al*., 2019). The Hi-C scaffolded assembly was polished with the 10X Genomics Illumina data by aligning to the assembly with longranger align, calling variants with freebayes ( Garrison & Marth, 2012). One round of the Illumina polishing was applied. The mitochondrial genome was assembled with MitoHiFi ( Uliano-Silva *et al*., 2021), which performed annotation using MitoFinder ( Allio *et al*., 2020). The assembly was checked for contamination as described previously ( Howe *et al*., 2021). Manual curation ( Howe *et al*., 2021) was performed using HiGlass ( Kerpedjiev *et al*., 2018) and Pretext. The genome was analysed within the BlobToolKit environment ( Challis *et al*., 2020). Table 3 contains a list of all software tool versions used, where appropriate.

**Table 3.** Software tools used.

Software tool	Version	Source
Hifiasm	0.14	Cheng *et al*., 2021
purge_dups	1.2.3	Guan *et al*., 2020
SALSA2	2.2	Ghurye *et al*., 2019
longranger align	2.2.2	https://support.10xgenomics.com/ genome-exome/software/pipelines/ latest/advanced/other-pipelines
freebayes	v1.3.1-17- gaa2ace8	Garrison & Marth, 2012
MitoHiFi	2	Uliano-Silva *et al*., 2021
HiGlass	1.11.6	Kerpedjiev *et al*., 2018
PretextView	0.2.x	https://github.com/wtsi-hpag/ PretextView
BlobToolKit	2.6.4	Challis *et al*., 2020

### Ethics/compliance issues

The materials that have contributed to this genome note have been supplied by a Darwin Tree of Life Partner. The submission of materials by a Darwin Tree of Life Partner is subject to the Darwin Tree of Life Project Sampling Code of Practice. By agreeing with and signing up to the Sampling Code of Practice, the Darwin Tree of Life Partner agrees they will meet the legal and ethical requirements and standards set out within this document in respect of all samples acquired for, and supplied to, the Darwin Tree of Life Project. Each transfer of samples is further undertaken according to a Research Collaboration Agreement or Material Transfer Agreement entered into by the Darwin Tree of Life Partner, Genome Research Limited (operating as the Wellcome Sanger Institute), and in some circumstances other Darwin Tree of Life collaborators.

## Data availability

European Nucleotide Archive: Seladonia tumulorum (bronze furrow bee). Accession number PRJEB45675; https://identifiers.org/ena.embl/PRJEB45675.

The genome sequence is released openly for reuse. The *S. tumulorum* genome sequencing initiative is part of the Darwin Tree of Life (DToL) project. All raw sequence data and the assembly have been deposited in INSDC databases. The genome will be annotated and presented through the Ensembl pipeline at the European Bioinformatics Institute. Raw data and assembly accession identifiers are reported in Table 1.

## Author information

Members of the University of Oxford and Wytham Woods Genome Acquisition Lab are listed here: https://doi.org/10.5281/zenodo.5746938.

Members of the Darwin Tree of Life Barcoding collective are listed here: https://doi.org/10.5281/zenodo.5744972.

Members of the Wellcome Sanger Institute Tree of Life programme are listed here: https://doi.org/10.5281/zenodo.6125027.

Members of Wellcome Sanger Institute Scientific Operations: DNA Pipelines collective are listed here: https://doi.org/10.5281/zenodo.5746904.

Members of the Tree of Life Core Informatics collective are listed here: https://doi.org/10.5281/zenodo.6125046.

Members of the Darwin Tree of Life Consortium are listed here: https://doi.org/10.5281/zenodo.5638618.
