# Supplementary material for: The genome sequence of the bronze furrow bee, Seladonia tumulorum (Linnaeus, 1758)
Source: Wellcome Open Res. 2025 Oct 1;7:105. Originally published 2022 Mar 21. [Version 2] doi: 10.12688/wellcomeopenres.17784.2 (PMC12511855; doi:10.12688/wellcomeopenres.17784.2)
Supplement: Peer review history

# Reviewer report: Reviewer response for version 2

**DOI:** 10.21956/wellcomeopenres.27569.r135028

**Published:** 2025-10-22

**Authors:** Fatih Dikmen

**Affiliations:** 1 Istanbul University, Istanbul, Turkey

This manuscript presents the high-quality, chromosome-level reference genome assembly of the **Bronze Furrow Bee, *Seladonia tumulorum***. This is a significant and valuable contribution to the field of hymenopteran genomics and general insect biology. The work was carried out as part of a major initiative (such as the Darwin Tree of Life project), which speaks to the high standard of the data generation and assembly methods used.

 The publication of the *S. tumulorum* reference genome is a **timely and essential contribution**. The technical quality of the assembly (large N50, chromosomal scaffolding) is fair enough. This resource will immediately support research in pollinator conservation, the evolution of social behavior in insects, and comparative genomics. I strongly recommend the acceptance of this manuscript, as it provides a fundamental dataset for the scientific community.

Are sufficient details of methods and materials provided to allow replication by others?

Yes

Is the rationale for creating the dataset(s) clearly described?

Yes

Are the datasets clearly presented in a useable and accessible format?

Yes

Are the protocols appropriate and is the work technically sound?

Yes

Reviewer Expertise:

Entomology, Insect genetics, taxonomy

I confirm that I have read this submission and believe that I have an appropriate level of expertise to confirm that it is of an acceptable scientific standard.

# Reviewer report: Reviewer response for version 2

**DOI:** 10.21956/wellcomeopenres.27569.r134804

**Published:** 2025-10-09

**Authors:** Barbara Feldmeyer

**Affiliations:** 1 Molecular Ecology Group, Senckenberg Biodiversity and Climate Research Centre (SBiK-F), Frankfurt, Germany

No more comments to the revised version.

Are sufficient details of methods and materials provided to allow replication by others?

No

Is the rationale for creating the dataset(s) clearly described?

Partly

Are the datasets clearly presented in a useable and accessible format?

Partly

Are the protocols appropriate and is the work technically sound?

Yes

Reviewer Expertise:

Evolutionary ecology, ecological genomics

I confirm that I have read this submission and believe that I have an appropriate level of expertise to confirm that it is of an acceptable scientific standard.

# Reviewer report: Reviewer response for version 1

**DOI:** 10.21956/wellcomeopenres.19683.r50085

**Published:** 2022-08-02

**Authors:** Lars Podsiadlowski

**Affiliations:** 1 Centre for Molecular Biodiversity Research, Zoological Research Museum Alexander Koenig (ZFMK), Bonn, Germany

This is a description of the genome of a halictide bee species. All the data is from the Darwin tree of life consortium. The usual metrics are provided in a table and graphical format (snail plot). All descriptions are concise and useful. The only thing that has to be changed: the assembly size given in the abstract differs from the one given in the text body and the table.

Are sufficient details of methods and materials provided to allow replication by others?

Yes

Is the rationale for creating the dataset(s) clearly described?

Yes

Are the datasets clearly presented in a useable and accessible format?

Yes

Are the protocols appropriate and is the work technically sound?

Yes

Reviewer Expertise:

Insect genomics, phylogenomics

I confirm that I have read this submission and believe that I have an appropriate level of expertise to confirm that it is of an acceptable scientific standard.

# Reviewer report: Reviewer response for version 1

**DOI:** 10.21956/wellcomeopenres.19683.r49748

**Published:** 2022-04-22

**Authors:** Barbara Feldmeyer

**Affiliations:** 1 Molecular Ecology Group, Senckenberg Biodiversity and Climate Research Centre (SBiK-F), Frankfurt, Germany

In the manuscript, the authors, Falk and Monks, present the genome of the bronze furrow bee as produced by utilising several PacBio, 10x, and Hi-C sequencing technologies. Below I detail some aspects which should be elaborated more or clarified. 

 Background

- Please add some information at the end of the paragraph stating the motivation for sequencing this species. This could include either some biological rationale, or that this species is part of DToL which aims to genetically describe all species in the UK.

Genome sequence report

- " *The genome was sequenced from a single male*" is misleading. One male was used for the assembly and another male for chromosome-scale scaffolding.
- " *A total of 58-fold coverage in Pacific Biosciences single-molecule long reads and 93-fold coverage in 10X Genomics read clouds were generated*." This information implies that the assembly was constructed using a mix of PacBio and 10x. Based on the "Genome assembly" paragraph it seems that 10x sequences were "only" used for polishing. Please be more specific.
- Does the 404 Mb length of the final assembly resemble the expected genome size? 
- The Hi-C contact map is rather messy. Could you either put more effort into manually curating the scaffolds or at least add some ideas and information on why the HiC scaffolding shows this ambiguous pattern?  

Methods

- The sample acquisition and DNA extraction sections are not very precise, please clarify.
- Unclear whether the " *Low molecular weight DNA was removed from a 200-ng aliquot of extracted DNA using 0.8X AMpure XP purification kit prior to 10X Chromium sequencing*" was only performed for the DNA sent to 10x sequencing or all HMW DNA.
- " *HMW DNA was sheared into an average fragment size between 12–20 kb in a Megaruptor 3 system with speed setting 30. Sheared DNA was purified by solid-phase reversible immobilization using AMPure PB beads with a 1.8X ratio of beads to sample to remove the shorter fragments and concentrate the DNA sample*." Unclear which part of the DNA was subjected to this protocol and what it was used for afterward.
- Information or link to the mitochondrial genome is missing.

Are sufficient details of methods and materials provided to allow replication by others?

No

Is the rationale for creating the dataset(s) clearly described?

Partly

Are the datasets clearly presented in a useable and accessible format?

Partly

Are the protocols appropriate and is the work technically sound?

Yes

Reviewer Expertise:

Evolutionary ecology, ecological genomics

I confirm that I have read this submission and believe that I have an appropriate level of expertise to confirm that it is of an acceptable scientific standard, however I have significant reservations, as outlined above.

# Response

**Published:** 2025-09-23

**Authors:** Tree of Life Team Sanger

**Affiliations:** Wellcome Trust Sanger Institute, UK

Thank you for reviewing this data note. Our responses to your comments are below.  

- Please add some information at the end of the paragraph stating the motivation for sequencing this species. This could include either some biological rationale, or that this species is part of DToL which aims to genetically describe all species in the UK.

*Response: We have added this text to the Background section. *

- " *The genome was sequenced from a single male*" is misleading. One male was used for the assembly and another male for chromosome-scale scaffolding.

*Response: Hi-C data were used only for linkage evidence to generate chromosome-scale scaffolds. No Hi-C reads were incorporated into contigs, and they were not used for polishing or gap filling. Thus the genome assembly sequence is from iySelTumu1 only.*  

- " *A total of 58-fold coverage in Pacific Biosciences single-molecule long reads and 93-fold coverage in 10X Genomics read clouds were generated*." This information implies that the assembly was constructed using a mix of PacBio and 10x. Based on the "Genome assembly" paragraph it seems that 10x sequences were "only" used for polishing. Please be more specific.

*Response: We have removed the short-read coverage phrase.*

- Does the 404 Mb length of the final assembly resemble the expected genome size? 

*Response: The genome length is within the expected range. The Merqury.FK completeness value of 95.6% which we have given in version of this data note shows that the assembly captures nearly all of the genome content.*  

- The Hi-C contact map is rather messy. Could you either put more effort into manually curating the scaffolds or at least add some ideas and information on why the HiC scaffolding shows this ambiguous pattern?  

*Response: The Hi-C contact map is messy due to the nature of the underlying data – hymenopteran genomes are often challenging due to the high number of repetitive sequences. We have supplied a labelled static chromosome map using PretextSnapshot.*   Methods

- The sample acquisition and DNA extraction sections are not very precise, please clarify.
- Unclear whether the " *Low molecular weight DNA was removed from a 200-ng aliquot of extracted DNA using 0.8X AMpure XP purification kit prior to 10X Chromium sequencing*" was only performed for the DNA sent to 10x sequencing or all HMW DNA.
- " *HMW DNA was sheared into an average fragment size between 12–20 kb in a Megaruptor 3 system with speed setting 30. Sheared DNA was purified by solid-phase reversible immobilization using AMPure PB beads with a 1.8X ratio of beads to sample to remove the shorter fragments and concentrate the DNA sample*." Unclear which part of the DNA was subjected to this protocol and what it was used for afterward.

*Response: We have edited the text to clarify that DNA was prepared for two different sequencing technologies.*

- Information or link to the mitochondrial genome is missing.

*Response: The accession number for the mitochondrial genome is already given in Table 2. We have now added it to the text as well: “The mitochondrial genome was also assembled (length 17.31 kb, OU565283.1).”* The Tree of Life team.
